# Agricultural intensification and the evolution of host specialism in the enteric pathogen *Campylobacter jejuni*

**DOI:** 10.1073/pnas.1917168117

**Published:** 2020-05-04

**Authors:** Evangelos Mourkas, Aidan J. Taylor, Guillaume Méric, Sion C. Bayliss, Ben Pascoe, Leonardos Mageiros, Jessica K. Calland, Matthew D. Hitchings, Anne Ridley, Ana Vidal, Ken J. Forbes, Norval J. C. Strachan, Craig T. Parker, Julian Parkhill, Keith A. Jolley, Alison J. Cody, Martin C. J. Maiden, David J. Kelly, Samuel K. Sheppard

**Affiliations:** ^a^The Milner Centre for Evolution, Department of Biology and Biochemistry, University of Bath, BA2 7AY Bath, United Kingdom;; ^b^Department of Molecular Biology and Biotechnology, The University of Sheffield, S10 2TN Sheffield, United Kingdom;; ^c^Cambridge Baker Systems Genomics Initiative, Baker Heart and Diabetes Institute, Melbourne, VIC 3004, Australia;; ^d^Department of Infectious Diseases, Central Clinical School, Monash University, Melbourne, VIC 3004, Australia;; ^e^Swansea University Medical School, Swansea University, SA2 8PP Swansea, United Kingdom;; ^f^Department of Bacteriology, Animal and Plant Health Agency-Weybridge, Addlestone, KT15 3NB Surrey, United Kingdom;; ^g^School of Medicine, Medical Sciences and Dentistry, University of Aberdeen, Foresterhill, AB25 2ZD Aberdeen, Scotland, United Kingdom;; ^h^School of Biological Sciences, University of Aberdeen, AB24 3UU Aberdeen, Scotland, United Kingdom;; ^i^Produce Safety and Microbiology Unit, Western Region Research Center, Agricultural Research Service, US Department of Agriculture, Albany, CA 94710;; ^j^Department of Veterinary Medicine, University of Cambridge, CB3 0ES Cambridge, United Kingdom;; ^k^Department of Zoology, University of Oxford, OX1 3PS Oxford, United Kingdom

**Keywords:** *Campylobacter*, genomics, adaptation, evolution, recombination

## Abstract

Modern agriculture has dramatically changed the distribution of animal species on Earth. Changes to host ecology have a major impact on the microbiota, potentially increasing the risk of zoonotic pathogens being transmitted to humans, but the impact of intensive livestock production on host-associated bacteria has rarely been studied. Here, we use large isolate collections and comparative genomics techniques, linked to phenotype studies, to understand the timescale and genomic adaptations associated with the proliferation of the most common food-born bacterial pathogen (*Campylobacter jejuni*) in the most prolific agricultural mammal (cattle). Our findings reveal the emergence of cattle specialist *C. jejuni* lineages from a background of host generalist strains that coincided with the dramatic rise in cattle numbers in the 20th century. Cattle adaptation was associated with horizontal gene transfer and significant gene gain and loss. This may be related to differences in host diet, anatomy, and physiology, leading to the proliferation of globally disseminated cattle specialists of major public health importance. This work highlights how genomic plasticity can allow important zoonotic pathogens to exploit altered niches in the face of anthropogenic change and provides information for mitigating some of the risks posed by modern agricultural systems.

## Significance

Human activities have had a profound effect on the Earth’s ecosystems and biodiversity. This is evident among livestock species, such as cattle, that now constitute more biomass than all wild mammals combined. For bacteria living within these animals, this massive host expansion represents an opportunity to proliferate and spread globally through trade networks. This carries significant risks as pathogenic species can potentially spread to humans, but little is known about how these zoonotic bacteria evolve to exploit the changing host niche space. Here, we explain the nature and timescale of cattle adaptation in the world’s most common enteric bacterial pathogen (*Campylobacter jejuni*), highlighting potential risks of intensive livestock production in pathogen emergence.

In the relatively short history since the second agricultural revolution (17th to 19th centuries), intensive farming practices have dramatically changed the distribution of plants and animals on Earth. Today, livestock account for 60% of all mammal biomass, with cattle being the most abundant (1), far surpassing the biomass of all wild mammals combined (4%). This radical anthropogenic ecological change carries significant risk, and in recent decades, escalating livestock numbers and global trade have been linked with the emergence of zoonotic diseases that pose a significant threat to both animal and human health (2–4).

The most abundant human bacterial pathogen found in cattle is *Campylobacter* (5). These organisms inhabit the gastrointestinal tract of many warm-blooded animals and are present in the feces of around 20% of cows at a concentration of ∼3 × 10^4^ cfu/g (6). The most frequently isolated species, *Campylobacter jejuni* and *Campylobacter coli*, are the leading cause of bacterial gastroenteritis in high income countries (7, 8). These organisms typically infect humans via consumption of contaminated meat and poultry (9), leading to widespread morbidity and, occasionally, mortality in vulnerable groups (7, 8). There are an estimated 1.5 billion cattle on Earth, each of which produces around 30 kg of manure daily (1). Therefore, around 3 × 10^17^ (300 quadrillion) *Campylobacter* are excreted by cattle into the environment every day. The sheer magnitude of shedding is clearly important in terms of direct environmental contamination (5, 10) and potential spillover into the human food chain. However, while much research has focused on the epidemiology of transient human infection (11), little is known about how intensive farming may establish new transmission cycles among reservoir hosts and influence the evolution of livestock-adapted strains.

Spillover between reservoir host species is exemplified in *Campylobacter* by the existence of strains that are regularly isolated from multiple host species (ecological generalists), indicating recent host transitions (12). While frequent host transition and genome plasticity in *Campylobacter* can favor ecological generalism (12), expansion into a new host species is typically thought to be accompanied by evolutionary specialization and gradual divergence from the ancestral population. This process may be expedited in intensive farming where frequent animal contact, high animal numbers, and low genetic diversity provide opportunities for pathogens to evolve (2–4, 13), potentially promoting the emergence and proliferation of specialist lineages that exploit the niche more effectively. Furthermore, as host animal numbers and colonization increase, so does the population size of the associated bacteria, enhancing the efficiency by which natural selection favors livestock-adapted strains. In the context of increasingly intensive farming practices and extensive global trade networks, the shifting nature of livestock pathogens presents a major public health threat. For example, around half a million confirmed annual cases of human campylobacteriosis in Europe are caused by the two most common cattle associated *C. jejuni* genotypes (sequence types, ST-61 and ST-42) (9, 14, 15), with the actual infection rate potentially much higher (16).

In this study, we carry out a population-genomic analysis of 1,198 *C. jejuni* isolates chosen to represent known diversity among reservoir host species in order to describe the nature and timescale of adaptation to cattle. The data reveal a dynamic pattern of genome evolution coinciding with the intensification of livestock farming, and explain key adaptive processes of gene loss and gain that allow strains to exploit the metabolic and nutritional environment of the cow gut and cope with different immune pressures. These evolutionary processes explain the emergence of one of the most common food-borne bacterial pathogens.

## Results

### Cattle Specialist *C. jejuni* Emerged from Host Generalist Ancestors.

**Fig. 1.**
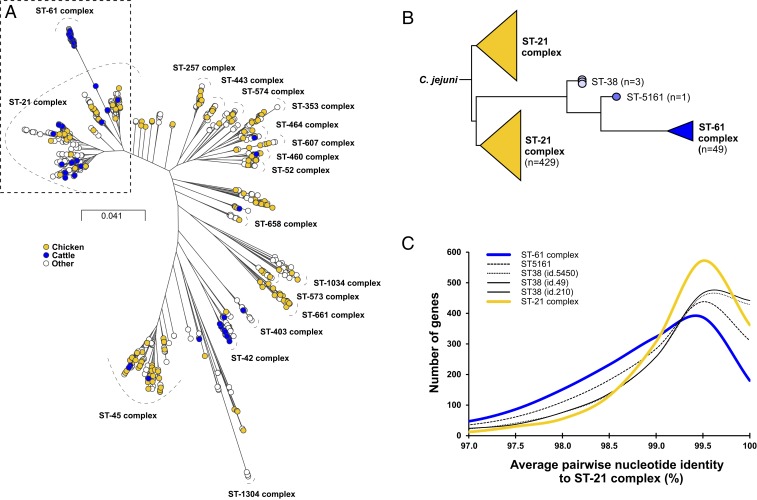
Cattle specialist *C. jejuni* emerged from a background of host generalists. (*A*) *C. jejuni* isolates (1,198) from chicken (yellow), ruminants (blue), and other sources (white) are shown on a phylogenetic tree reconstructed using an approximation of the ML algorithm implemented in RAxML, with the major MLST clonal complexes indicated next to the associated genome sequence cluster. The cattle specialist ST-61 clonal complex can be seen emerging from the generalist ST-21 complex (dashed box). The scale bar indicates the estimated number of substitutions per site. (*B*) ML tree of the branch differentiating ST-61 complex (blue) from ST-21 complex (yellow) isolates highlighting the existence of intermediate isolates (different shades of blue) indicative of step-wise cattle specialization. (*C*) Average nucleotide identity for pairwise comparisons of 1,208 core and 290 soft core genes for 46 genomes of ST-21 complex (yellow), 49 genomes of ST-61 complex (blue), and 4 intermediate isolates from ST-38 and ST-5161 (black line, black dotted, and black dotted, respectively).

*C. jejuni* strains representing the breadth of the known genotype and host-source diversity (Dataset S1) included 1,198 isolates from 18 different sources and belonging to 36 clonal complexes based upon sharing of four or more alleles at seven housekeeping gene loci defined by multilocus sequence typing (17). A maximum-likelihood (ML) phylogenetic tree based on the gene-by-gene sequence alignment of 1,418 genes present in >90% of isolates revealed genome sequence clusters that broadly corresponded to multilocus sequence typing (MLST) clonal complexes, consistent with previous studies (Fig. 1*A*) (14, 16, 18). The phylogeny indicated broad diversity of isolates with evidence of host-specialist (chicken or cattle) and host-generalist lineages (e.g., ST-21 and ST-45 complexes) (14, 18) (Fig. 1*A*). Consistent with host switching, isolates from cattle were common in generalist clades, but 36% were found in discrete cattle-associated clusters (ST-61 and ST-42 complexes). Strikingly, most ST-61 complex isolates (98%) formed a tight cluster at the tip of a longer branch emanating from within the ST-21 complex (Fig. 1 *A* and *B*), potentially indicative of rapid differentiation from the ancestral population. To support evidence of the rapid expansion of this lineage, we calculated Tajima’s D for the alignment of ST-61 complex isolate genomes (19, 20). This generated a negative value (−1.569), implying an abundance of low-frequency polymorphisms, consistent with rapid clonal expansion of the population. Demographic reconstruction analysis using BEAST2 (21) showed an increase in effective population size consistent with a scenario of rapid expansion of this lineage (*SI Appendix*, Fig. S1). Comparable analysis performed on ST-42 complex isolates showed no evidence of rapid lineage expansion based on Tajima’s D (0.2) or demographic reconstruction (*SI Appendix*, Fig. S1).

Four isolates from cattle represented intermediates between the ST-21 and ST-61 complex sequence clusters (Fig. 1*B*). Comparison of the allelic variation in 43 genes that showed >90% allele segregation by complex (*SI Appendix*, Fig. S2) revealed a gradual increase in “ST-61–like” alleles from the first intermediate isolates (ST-38, 11/43) to the second intermediate (ST-5161, 27/43). The frequency distribution of average nucleotide identities for 1,208 core and 290 soft core ST-61 and ST-21 complex genes in the intermediate isolates revealed gradual differentiation from the ancestral ST-21 population, with a median core genome nucleotide identity decreasing from 99.47 (ST-38 isolates) to 99.25 (ST-5161 isolate) (Fig. 1 *B* and *C*). These findings are consistent with the step-wise emergence of a cattle-associated lineage.

### Intensive Cattle Farming Coincides with the Emergence of Host Specialist *C. jejuni*.

**Fig. 2.**
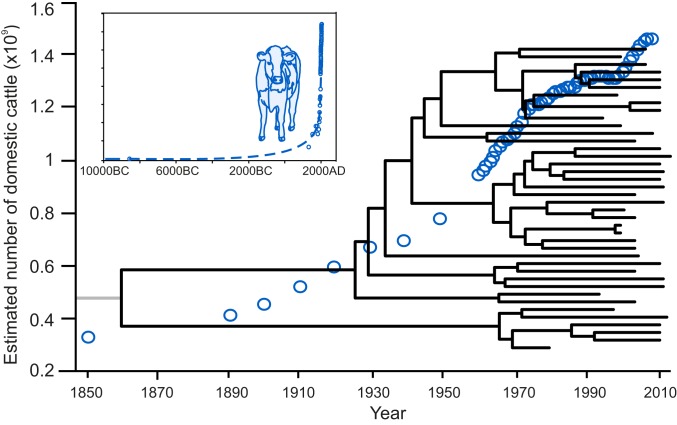
The emergence of cattle specialist *C. jejuni* coincides with modern intensive livestock farming. Graph of the estimated number of domestic cattle (blue circles) on Earth from 1850 to 2010 (*Inset* box from 10000 BC to 2000 AD) based upon data from refs. 24–27. Time-scaled phylogeny of 41 *C. jejuni* isolates showing dating estimates of the ST-61 complex emergence with ST-5161 as an outgroup, indicated as a gray line. The MRCA of ST-61 and ST-5161 was estimated.

To estimate the age of the emergence of the most common cattle specialist lineage, we used 41 ST-61 complex isolates with known isolation date ranging from 1979 to 2013. A 1,490,602-bp core genome alignment of the 41 ST-61 complex isolates (90.81% of the NCTC11168 *C. jejuni* reference genome) was ordered on NCTC11168. Inferred recombinant regions were stringently masked to recover the population clonal frame and improve the timed-measured approximation of the phylogeny. The temporal signal of the phylogeny was calculated using TempEst v1.5.1 (22) (correlation coefficient ranged from 0.411 to 0.614) for ST-61 and ST-42 complexes and was consistent with other estimates for *C. jejuni* (23). After removal of the recombined regions, only variable sites remained. Ancestral dates were estimated for internal nodes using the tip dates of the ST-61 phylogeny and using a relaxed log normal clock model in BEAST2 (21). The dated phylogeny approximated the emergence of the ST-61 complex to have occurred in 1859 (95% Highest Posterior Density: 1692–1943) (Fig. 2). Much of the ST-61 sublineage diversification was more recent (1925–1965), and there was evidence that population expansion coincided with the intensification of cattle farming throughout the 20th century. The same analysis was replicated for ST-42 complex where the time to most recent common ancestor was dated in 1514 (95% Highest Posterior Density: 6647 BC–1943 AD) (*SI Appendix*, Fig. S1). Sublineages within the ST-42 complex have emerged from 1629 to 1954 (*SI Appendix*, Fig. S1).

### Accessory Gene Gain Is Associated with Cattle Specialization.

**Fig. 3.**
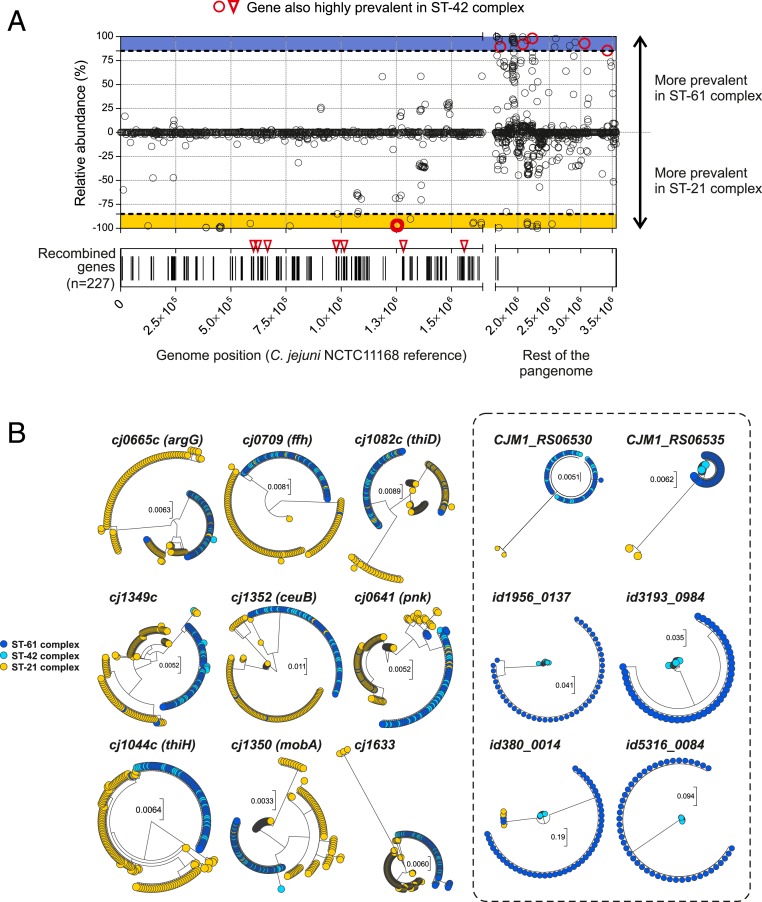
The cattle specialist lineage genomes show evidence of host adaptation. (*A*) Gene presence/absence (black circles) and recombination regions (black bars) gained and lost on the tree branch differentiating ST-61 complex cattle specialist *C. jejuni* inferred using ClonalFrameML and mapped to the NCTC11168 reference genome. Accessory genes not mapped to NCTC11168 are also highlighted on the left (black circles, *Upper*). The relative abundance of genes was calculated as presence in ST-61 complex isolates minus presence in ST-21 complex isolates. The frequency of all genes/alleles (black circles/bars) is shown for ST-61 and ST-21 complexes (*Upper* and *Lower*). The homoplasic genes (red circles), including the glycosylation gene block, and recombinant alleles (red triangles) present or absent in ST-42 complex are shown (*Upper* and *Lower*). (*B*) Single-gene trees for nine candidate cattle-adaptive genes demonstrating homoplasy among cattle specialist lineages. Trees show intermingled clusters of isolates from the two cattle specialist lineages ST-61 (blue) and ST-42 (light blue), separated from the generalist lineage ST-21 (yellow).

Both gene gain and gene loss can contribute to pathogen evolution and host tropism (28, 29), and this has been observed in *Campylobacter* (18, 30). Comparative genomic analysis revealed 1,225 core genes present in all 1,198 isolates and 2,629 accessory genes that were differentially present. The prevalence of accessory genes varied between lineages (*SI Appendix*, Fig. S3) and was correlated with isolate host source. For example, a total of 35 genes were present in >90% of all ST-61 complex isolates (*n* = 44/49) but absent in >90% of all ST-21 complex isolates (*n* = 7/113) (Fig. 3*A* and Dataset S2). This analysis was also performed to compare ST-42 and ST-21 complex isolates (Dataset S3). While this does not prove that these genes confer an advantage when colonizing cattle, they can be considered to represent candidate adaptive genes. Most cattle-associated accessory genes were annotated as hypothetical proteins of unknown function due to a lack of homology with clearly annotated and well-characterized genes from an appropriate laboratory reference strain (31). Where the function could be inferred, genes were related to motility-associated factors (*maf* family genes), involved in flagellar biosynthesis (*cj1340* and *cj1341c*) (32, 33) and capsule biosynthesis (*id86_0802* and *id86_0803*) (Table 1 and Dataset S2). Flagellar motility and surface structures are known to impact colonization of animals in vivo (34–36), providing some support for a possible adaptive role for these genes in cattle colonization. However, in vivo assays would be necessary to confirm this.

**Table 1.** Candidate adaptive genes involved in ST-61 complex emergence from ST-21 clonal complex

Name	Alias	Transcriptional unit no.*	Genome position on *C. jejuni* NCTC11168 reference genome	Predicted function^†^	COG family ^†^	COG family description^†^	Prevalence in ST-21 complex, *n* = 113 (%)	Prevalence in ST-61 complex, *n* = 49 (%)	Prevalence in ST-42 complex, *n* = 22 (%)
Accessory genes highly prevalent in ST-61 and ST-42 complexes but missing in ST-21 complex									
* CJM1_RS06530*	—	—	—	Hypothetical protein	—	—	2 (1.77)	44 (89.80)	18 (81.82)
* CJM1_RS06535*	—	—	—	Plasmid stabilization system protein	R	General function prediction only	2 (1.77)	44 (89.80)	18 (81.82)
* id1956_0137*	*cj1340-like*	—	—	Motility accessory factor (homolog)	S	Function unknown	0 (0)	45 (91.84)	21 (95.45)
* id3193_0984*	*cj1341-like*	—	—	Motility accessory factor (homolog)	S	Function unknown	0 (0)	44 (89.80)	20 (90.91)
* id380_0014*	—	—	—	Hypothetical protein	—	—	4 (3.54)	48 (97.96)	22 (100)
* id5316_0084*	—	—	—	Putative McrB subunit of the McrBC restriction endonuclease system	—	—	0 (0)	48 (97.96)	15 (68.18)
Accessory genes highly prevalent in ST-21 complex but missing in ST-61 and ST-42 complexes									
* Cj1319*	—	495	1248624..1249595	Putative nucleotide sugar dehydratase	M	Cell wall/membrane biogenesis	113 (100)	1 (2.04)	0 (0)
* Cj1320*	—	495	1249588..1250742	Putative DegT family aminotransferase	M	Cell wall/membrane biogenesis	113 (100)	2 (4.08)	0 (0)
* Cj1324*	—	498	1252278..1253399	Hypothetical protein	D	Cell cycle control, mitosis and meiosis	112 (99.1)	1 (2.04)	0 (0)
* Cj1325*	—	498	1253417..1254092	Putative methyltransferase	J	Translation	112 (99.1)	1 (2.04)	0 (0)
* Cj1327*	*neuB2*	498	1254132..1255136	*N*-acetylneuraminic acid synthetase	M	Cell wall/membrane biogenesis	113 (100)	2 (4.08)	0 (0)
* cj1328*	*neuC2*	498	1255129..1256283	Putative UDP-*N*-acetylglucosamine 2-epimerase	M	Cell wall/membrane biogenesis	113 (100)	1 (2.04)	0 (0)
* cj1329*	—	498	1256292..1257317	Putative sugar-phosphate nucleotide transferase	J	Translation	113 (100)	1 (2.04)	0 (0)
* cj1330*	—	498	1257314..1258219	Hypothetical protein	R	General function prediction only	113 (100)	1 (2.04)	0 (0)
* cj1331*	*ptmB*	498	1258212..1258919	Acylneuraminate cytidylyltransferase	M	Cell wall/membrane biogenesis	113 (100)	2 (4.08)	0 (0)
* cj1332*	*ptmA*	498	1258919..1259689	Flagellin modification protein A	I	Lipid transport and metabolism	113 (100)	2 (4.08)	0 (0)
Alleles of core genes harboring recombination events highly prevalent in ST-61 and ST-42 complexes but missing in ST-21 complex									
* cj0641*	*pnk*	243	602326..603186	Inorganic polyphosphate/ATP-NAD kinase	G	Carbohydrate transport and metabolism	4 (3.54)	39 (79.59)	20 (90.91)
* cj0665c*	*argG*	248	621392..622612	Argininosuccinate synthase	E	Amino acid transport and metabolism	4 (3.54)	48 (97.96)	21 (95.45)
* cj0709*	*ffh*	269	665788..667125	Signal recognition particle protein	U	Intracellular trafficking and secretion	4 (3.54)	49 (100)	20 (90.91)
* cj1044c*	*thiH*	395	977626..978771	Thiamine biosynthesis protein ThiH	H	Coenzyme transport and metabolism	0 (0)	49 (100)	22 (100)
* cj1082c*	*thiD*	405	1013374..1014186	Phosphomethylpyrimidine kinase	H	Coenzyme transport and metabolism	6 (5.31)	39 (79.59)	22 (100)
* cj1349c*	—	500	1280992..1282299	Putative fibronectin/fibrinogen-binding protein	K	Transcription	0 (0)	47 (95.92)	17 (77.27)
* cj1350*	*mobA*	501	1282361..1282936	Putative molybdopterin-guanine dinucleotide biosynthesis protein	H	Coenzyme transport and metabolism	3 (2.65)	42 (85.71)	19 (86.36)
* cj1352*	*ceuB*	502	1284008..1284976	Enterochelin uptake permease	P	Inorganic ion transport and metabolism	3 (2.65)	48 (97.96)	22 (100)
* cj1633*	—	601	1558654..1559637	Putative ATP-binding protein	J	Translation	0 (0)	44 (89.80)	21 (95.45)

ST-38 and ST-5161 intermediate isolates were not included in the prevalence calculations. One-letter abbreviations are linked to the functional categories in column 7 as defined by the Clusters of Orthologous Groups (COG) classification (37).

* As predicted by the Prokaryotic Operon DataBase (ProOpDB) (38). Consecutive numbers reflect consecutive transcriptional units on the *C. jejuni* NCTC11168 reference genome.

^†^ As predicted by the WebMGA server (39).

### Homologous Recombination and Cattle Specialization.

A broadly accepted evolutionary principle in microbiology is that host-associated bacterial lineages with reduced genomes typically derive from ancestors with larger genomes (40), because of mutational bias toward deleting superfluous or redundant genes (41). There is also evidence that genome reduction is enhanced when there is isolation from the ancestral population so recombination cannot mitigate against the accumulation of deleterious mutations (42). There was no evidence of genome reduction among ST-61 complex isolates sampled from 1979 to 2013 (*SI Appendix*, Fig. S4) or in the complex as a whole (1,527–1,835 genes) compared to ST-21 (1,527–1,885 genes), but this could be because of the relatively recent emergence of this cattle-associated lineage. To investigate the extent of gene pool isolation, we quantified the extent of allele sharing (1,308 core genes) for 220 isolate genomes from clonal complexes that have been sampled from both chickens and cattle. In six of nine clonal complexes, the average number of ST-61 alleles in the core genome was higher in isolates from cattle compared to those from chickens (*SI Appendix*, Fig. S5). This is consistent with homologous recombination among lineages that cohabit in cattle.

A more detailed analysis of homologous recombination across the genome was carried out using ClonalFrameML to identify putative recombinant sites associated with the emergence of cattle specialist *C. jejuni.* Recombination had a considerable role in generating genetic diversity within the ST-61 clonal complex, leading to 12.6 times as many nucleotide substitutions as mutation compared to a recombination to mutation ratio (*r*/*m*) of 7.5 for the generalist ST-21 complex and 9.6 for the entire dataset (*SI Appendix*, Table S1). These estimates are higher than the most widely cited *r/m* estimate for *C. jejuni* (2.2) (43) that is based on seven MLST genes (44). However, for recombining bacteria, *r*/*m* estimates can vary widely even among lineages within species (45, 46). Furthermore, recombination is likely to play a lesser role on the highly conserved MLST loci than it does throughout the genome. Our results suggest that recombination is associated with the emergence of the ST-61 complex and the process of cattle specialization. A total of 5,019 recombination events were recorded (*SI Appendix*, Fig. S6), of which 175 occurred on the tree branch separating ST-61 from ST-21 complex isolates. These recombination events mapped to 227 genes including 42 whole-gene replacements and 185 mosaic genes (Dataset S2). Unconfirmed clues about the possible adaptive role of recombination come from the inferred function of recombinant genes. A total of 29.8% were related to metabolic functions, 10.5% were related to cell envelope biogenesis, and 22.9% were known flagella genes linked to cell motility (Dataset S2). Alterations to flagella are known to be important for colonization of the host environment (47), particularly in *C. jejuni* and *Helicobacter pylori*, where flagellar motility is a key adaptive trait enabling bacteria to move effectively through viscous environments such as the lumen and mucus layer of the gastrointestinal tract (48). Similar analysis on ST-42 complex inferred 184 recombination events, mapping to 621 genes, 102 of which were also found to have recombined on the branch leading to the ST-61 complex (Dataset S3).

### Convergent Evolution Reveals Adaptive Genomic Changes.

It can be challenging to differentiate adaptive genomic changes that provide an advantage in the host from changes resulting from genetic bottlenecks and drift (42). Among the most compelling evidence for genome adaptation occurs when divergent lineages independently acquire convergent genetic changes, which are not present in their common ancestor (homoplasy). Preliminary automated analysis of homoplasy (49) identified variation in the number of homoplasies among clonal complexes (*SI Appendix*, Fig. S7). We then conducted more detailed analysis, consistent with studies identifying chicken adaptation in *Staphylococcus aureus* (50), to identify homoplasy between phylogenetically distant cattle specialist ST-61 and ST-42 complexes. Hypothesizing that differential gene prevalence in host-associated lineages is evidence of adaptation, we first identified 326 genes that were statistically associated with either ST-61 or ST-21 complexes (Fisher’s exact test, *P* < 0.0005). Focusing on genes that were present at >90% in one complex and <10% in the other, we identified 35 genes that were overrepresented (Fisher’s exact test, *P* < 10*e*-13) in the ST-61 complex and 39 genes that were largely absent. We also identified 175 inferred recombination blocks (mapping to 227 genes) on the branch leading to the ST-61 complex. The presence/absence of these elements was investigated in ST-42 and 12 other clonal complexes to identify homoplasy (Dataset S2).

**Fig. 4.**
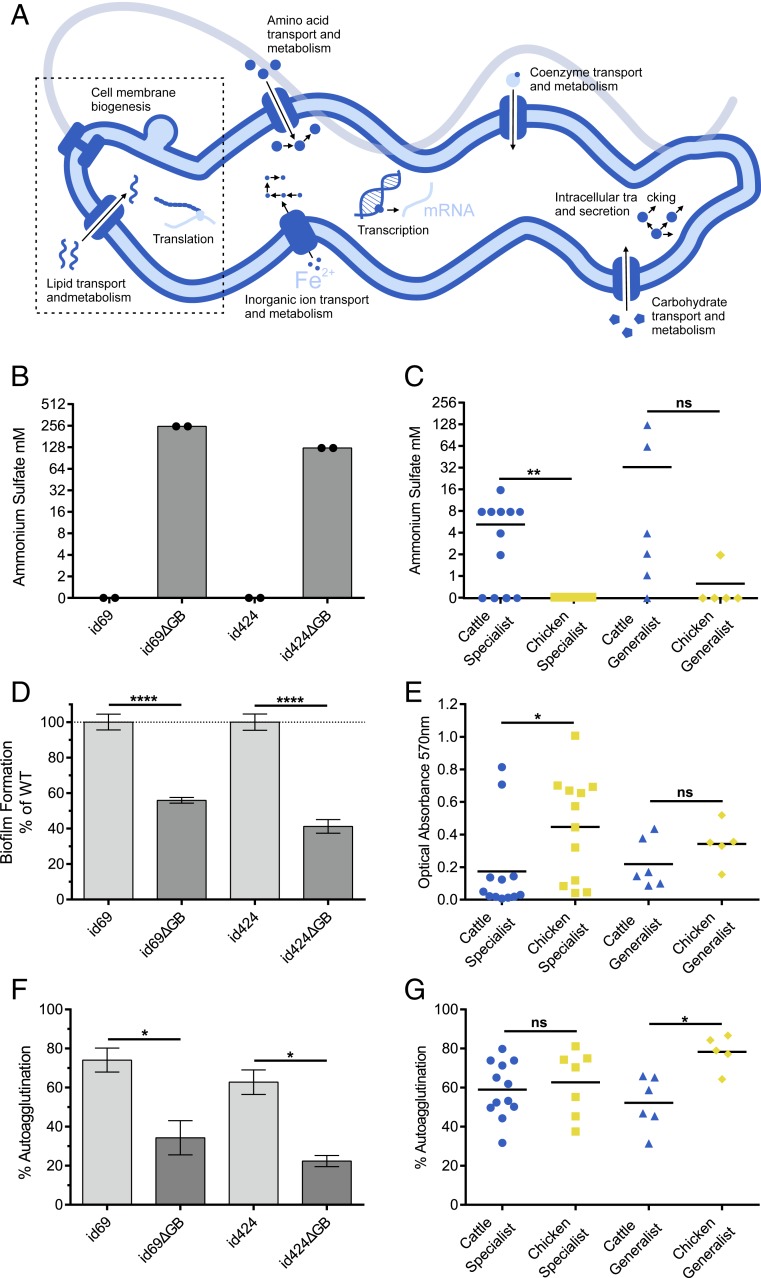
Evidence of cattle associated phenotype variation in *C. jejuni*. (*A*) Summary of cattle adaptation based on comparative genomics indicating biological pathways associated with deletion of the glycosylation gene block. (*B*–*G*) Phenotype assays comparing two WT chicken specialist strains, id69 and id424 (Dataset S1) and isogenic glycosylation gene block deletion mutants (id69ΔGB and id424ΔGB), and natural specialist and generalist strains from cattle and chicken. (*B*, *D*, and *F*) Chicken-associated lineage mutants show a marked decrease in cellular hydrophobicity, biofilm formation, and autoagglutination compared to WT strains. In assays of natural strain collections, cattle specialist strains show significantly greater mean ammonium sulfate concentration (millimolar) indicating decreased cell hydrophobicity (*C*), and significantly decreased ability to from biofilms measured as optical absorbance (at 570 nm) (*E*). (*G*) No difference was observed in autoagglutination in specialist strains from cattle and chicken, but generalist strains from cattle showed decreased ability to autoagglutinate. Significance was tested using Mann–Whitney *U* test: *P < 0.05, **P < 0.01, and ****P < 0.001. The horizontal line in each plot represents the mean value.

Of the ST-61 complex-associated genes (*n* = 35) and recombination blocks (*n* = 175), we identified six genes and nine recombinant alleles (respectively) that were also present in ST-42 isolates (Fig. 3*B*) and were largely absent in ST-21 complex isolates and chicken-specialist ST-257, ST-353, ST-443, ST-573, ST-574, ST-607, and ST-661 complexes (Table 1 and *SI Appendix*, Figs. S8 and S9). Accessory genome homoplasy included homologs of genes *cj1340* and *cj1341* from the *maf* gene family encoding flagella biosynthesis and bacterial motility factor proteins (32, 33). Recombinant genes with homoplasious alleles included six genes (*pnk*, *argG*, *thiH*, *thiD*, *mobA*, *ceuB*) associated with metabolism or transport, including genes involved in thiamine biosynthesis (*thiH* and *thiD*), molybdenum cofactor biosynthesis (*mobA*) for respiratory enzymes, and a putative fibronectin/fibrinogen-binding protein gene (*cj1349*) involved in host cell adhesion (Table 1 and Fig. 4*A*).

### Accessory Gene Loss Is Linked to Cattle Adaptation.

A striking finding was the identification of gene blocks, including 10 genes, that were largely missing in ST-61 (*n* = 48/49) and ST-42 (*n* = 22/22) complex isolates but present in all ST-21 and other chicken-associated complexes (Table 1, *SI Appendix*, Figs. S8 and S9, and Dataset S2). These genes were colocated in the genome; organized into two operons, suggesting functional linkage (Table 1); and included *cj1319, cj1320, cj1324*, *cj1325*, *cj1327* (*neuB2*), *cj1328* (*neuC2*), *cj1329*, *cj1330*, *cj1331* (*ptmB*), and *cj1332* (*ptmA*), all located in a region encoding the flagellin O-linked glycosylation system. Genes *cj1324* and *cj1325* encode enzymes necessary for the biosynthesis of legionaminic acid, a surface-exposed polysaccharide that has been shown in previous studies to be essential for chicken colonization (51) (Table 1). Furthermore, genes *cj1331* (*ptmB*) and *cj1332* (*ptmA*) have been shown to be involved in posttranslational modification of the flagellin protein first described in *C. coli* VC167 strain (Table 1) (52). The independent loss of these genes since the emergence of the two major cattle specialist lineages is not consistent with a random bottlenecking event associated with colonization and may indicate selection against strains that carry superfluous genes (beneficial in chicken and not cattle) because of a fitness cost to the bacterium (28–30). Similar analysis for ST-42 complex revealed the loss of 34 genes, all of which were also lost in ST-61 complex (Dataset S3), but in vivo colonization assays would be necessary to confirm the adaptive advantage of gene loss.

### Cattle Adaptation Is Associated with Functional Variation in Cell Hydrophobicity, Autoagglutination, and Biofilm Formation.

Identification of homplasious genomic signatures provides broad information about adaptive evolution but focusing on specific groups of genes, such as the lost glycosylation gene block, provides information about the functional basis of phenotypic adaptation. To investigate this, phenotype assays were conducted in vitro on 36 *C. jejuni* strains from cattle specialist (ST-61 and ST-42 complex), generalist (ST-21 complex), and chicken specialist lineages, where the glycosylation gene block was differentially present, and two engineered ΔGB (gene block) mutant strains (ST-661 and ST-257 complex) where the glycosylation gene block (*cj1324-1332*) was specifically deleted by allelic exchange mutagenesis.

Posttranslational modifications of flagellin are known to be important in autoagglutination and hydrophobicity (32, 53), and this is associated with virulence in Gram-negative bacteria (54), including *Neisseria gonorrhoeae* (55) and *Bordetella pertussis* (56). The engineered ΔGB mutant strains both displayed a dramatic loss of hydrophobicity, measured as an increase in the salt concentration at which cell aggregation occurs, compared to the otherwise isogenic WT parental strains (Fig. 4*B*). Furthermore, *C. jejuni* strains from cattle specialist lineages displayed a significant reduction in hydrophobicity compared to those from chicken specialist lineages (Mann–Whitney *U* test, *P* = 0.013) (Fig. 4*C*). In the generalist ST-21 lineage, isolates from cattle showed a trend toward reduced hydrophobicity compared to those from chicken, although not significant (Fig. 4*C*). Similarly, autoagglutination was significantly reduced in the engineered ΔGB mutant strains compared to the isogenic WT parents (unpaired *t* test, *P* = 0.0343 and *P* = 0.0142) (Fig. 4*F*). A significant reduction was also observed among ST-21 complex generalist isolates from cattle compared to those from chickens (Mann–Whitney *U* test, *P* = 0.0173) and a nonsignificant reduction was observed in cattle specialist strains compared to the chicken specialists (Fig. 4*G*).

Biofilm formation has also been strongly linked with flagellar properties and function (57). Consistent with this, a highly significant reduction in biofilm formation was observed in the ΔGB mutants compared to the WT parental strains (unpaired *t* test, *P* < 0.0001) (Fig. 4*D*). Similarly, chicken specialist isolates displayed enhanced biofilm formation compared to cattle specialist strains (Mann–Whitney *U* test, *P* = 0.0387) (Fig. 4*E*). For the generalist ST-21 lineage, strains originating from chicken showed a higher average ability to form biofilm compared to strains from cattle, although the difference was not significant (Fig. 4*E*). There was no significant difference in biofilm formation between strains of ST-61 and ST-42 cattle specialist lineages.

## Discussion

The well-established link between anthropogenic environmental change and the emergence of zoonoses usually focuses on pathogen spill-over and spread in new host populations (13, 58). However, for multihost pathogens, changes to the host niche can also have a major impact on preexisting transmission networks, enhancing the risk of human infection. Undoubtedly the most dramatic change to the natural host niche of *C. jejuni* has occurred with the advent of intensive livestock production favoring sublineages associated with agricultural animal hosts, such as cattle (16, 18, 30). This affords the opportunity to investigate the genomics and timescale of host adaptation in the industrialised world’s most common food-borne bacterial pathogen.

Host generalist *C. jejuni* lineages, containing isolates sampled from both chicken and cattle, are indicative of multiple recent host transition events (Fig. 1*A*). We demonstrate that against this dynamic backdrop of mingling bacterial populations, a cattle-associated lineage—the ST-61 complex—arose and proliferated. This process of host specialization, in apparent sympatry, may have proceeded with the gradual step-wise differentiation from the generalist ST-21 complex ancestor, as exemplified by the existence of rare intermediate genotypes (Fig. 1*B*), ultimately leading to the emergence of successful endemic and epidemic clones circulating globally within cattle. In comparative analysis of the cattle-associated ST-42 complex, we could not confirm emergence from ecological generalist ancestors.

Dating the emergence of cattle specialist *C. jejuni* provides clues about the influence of livestock intensification on pathogen evolution. Cattle were first domesticated from wild aurochs (*Bos primigenius*) in the early Neolithic age (59). Based upon its distribution in wild ungulates (60, 61) it is likely that *Campylobacter* was present in some cattle throughout the 10,000-y history of domestication. However, the emergence of the modern cattle specialist ST-61 lineage was dated much more recently, in the mid-to-late 1800s, with most lineages emerging in the 20th century. This is consistent with the rapid evolution of cattle specialism coinciding with the industrial agriculture revolution and the intensification of animal production (62, 63). Between 1820 and 1975, the number of cattle increased eightfold across the world (64–66). New farming techniques, improved livestock breeding, and higher stocking densities led to amplified food production, but the host population explosion and global transmission networks have also fundamentally changed the cattle niche, potentially favoring strains that are adapted to specialist lineages.

Horizontal gene transfer (HGT) plays an important role in the evolution of *Campylobacter*, generating genetic diversity at twice the rate of de novo mutation (44) and potentially conferring novel function on the recipient genome. Characterization of segments of DNA that have recombined in cattle specialists provided information about the host-specific gene pool. Furthermore, to differentiate host-adaptive genetic signatures from host associations that may reflect bottlenecking and drift from the ancestral gene pool, we focused on homoplasious genomic changes that occurred independently in divergent cattle specialist lineages. Our analysis revealed combinations of genes and alleles that potentially promote the host-adaptive evolution and specialization in divergent lineages.

Cattle and poultry display clear differences in anatomy, physiology, and metabolism. Consistent with this, candidate cattle-adaptive genetic variation occurred in genes involved in diverse functions. For example, the putative fibronectin/fibrinogen-binding protein gene *cj1349c* is important for in vitro adherence to chicken epithelial cells (67), therefore anatomic and histological differences with cattle intestinal epithelial cells may explain the conservation of alleles in cattle specialist *C. jejuni*. Furthermore, differences in the diet of cattle and chicken may account for signatures of cattle adaptation in the *C. jejuni* genome. Vitamin B1 supplements are commonly given in poultry feed at high doses (68) while comparable host requirements are synthesized naturally in cattle by rumen bacteria, where the majority is absorbed in the small intestine with only low amounts reaching the large intestine (69). This potentially means that *C. jejuni* in the cattle large intestine have distinct thiamine synthesis requirements accounting for homoplasy among thiamine biosynthesis genes (*thiH, thiD, thiE*) in cattle specialist *C. jejuni*. Similarly, the conservation of the *mobA* gene that encodes a key molybdopterin-guanine dinucleotide biosynthesis protein that allows *C. jejuni* to use a range of alternative electron donors and acceptors for respiration may help balance the rate of biosynthesis with the varying supply of potentially toxic (to cattle) molybdenum (70, 71). While speculations about differences in host anatomy and physiology provide a context for considering the putative function of cattle-associated genetic variation, in vivo growth assays would be necessary to confirm adaptation.

The emergence of cattle specialist *C. jejuni* was also associated with significant gene loss. While reductive evolution is widely observed among bacteria within discrete niches (72), in most cases it is not directly adaptive but results from drift or linkage to other beneficial mutations (42). However, in *Campylobacter* and *Salmonella enterica*, loss of gene function has been shown to be a characteristic of some host-restricted lineages (28–30, 73). Consistent with this, we observed distinct gene loss signatures in the flagellin O-linked glycosylation system in cattle-associated *C. jejuni,* including specialist lineages. This was associated with reductions in cell hydrophobicity, autoagglutination, and biofilm formation in comparisons among wild-type cattle and chicken specialist lineages and confirmed by studies with deletion mutants as controls. The flagellin protein is posttranslationally modified on the surface of the flagellum, which is exposed to the extracellular/host environment, and this is known to be associated with serospecificity (74), with O-linked glycosylation system genes overrepresented in isolates from chicken compared to cattle (51). The absence of genes in this gene block, and the associated changes in cell surface charge and glycan moieties on the flagellum (51), may enable *C. jejuni* to attach better to the intestinal epithelium as changes in hydrophobicity in the flagella are believed to enable bacterial attachment to either hydrophobic or hydrophilic surfaces (57). Furthermore, surface structures such as flagella are known to stimulate host innate and adaptive immunity (75). Therefore, the loss of the glycosylation gene block, and associated phenotypic changes, may decrease recognition of strains by the host–immune response. This provides a compelling basis for confirmatory in vivo experiments to investigate how gene loss may potentially influence proliferation in cattle.

Industrialized agriculture, including intensive livestock production, remains important to meet the nutritional requirements of a growing human population. However, the impact on livestock-associated pathogens is seldom considered. Our findings suggest a change to the evolutionary history of *C. jejuni* that preceded the modern agricultural revolution. As the vast global cattle niche opened in the last century, a host generalist lifestyle ceased to be the only effective strategy for cattle colonization and, thus, specialist strains emerged—facilitated by HGT and significant gene gain and loss, related to differences in host diet, anatomy, and histology—and were disseminated across the world. This highlights how the genomic plasticity of important zoonotic pathogens allows a response to radical anthropogenic changes to host ecology, potentially enhancing the risk of human infection. Further understanding of the genetic and functional basis of host adaptation, particularly to livestock that constitute the majority of mammal biomass on Earth, is important for the development of novel strategies, interventions, and therapies to combat the increasing risk of pathogens with the capacity to spread from livestock to humans.

## Methods and Materials

### Bacterial Isolates.

*C. jejuni* genome sequences were analyzed for 1,198 isolates sampled from clinical cases of campylobacteriosis, chickens, ruminants, environmental sources, pets, and wild birds from different countries (Dataset S1) (14, 16, 18, 30). These included 1,065 from published studies, 119 isolate genomes available on National Center for Biotechnology Information (NCBI) and 14 ST-61 complex isolates obtained from Animal and Plant Health Agency collections and sequenced as part of this study (Dataset S1). These included isolates from cattle (*n* = 8), sheep (*n* = 3), chicken (*n* = 1), giraffes (*n* = 1), and humans (*n* = 1), sampled 1993–2003 (Dataset S1). Comparative genomics centered on 101 ruminant isolates, principally cattle (*n* = 93), and 1,097 isolates from other sources, representing multiple lineages defined by multilocus sequence typing. These included 26 and 10 isolates from the cattle-associated ST-61 and ST-42 clonal complexes, respectively, sampled principally from the United Kingdom (60%), Spain (12%), and the United States (10%) (Dataset S1).

### Culture, DNA Extraction, and Genome Sequencing.

*C. jejuni* strains were cultured on Columbia base agar plates containing 5% (vol/vol) horse blood and 5 µg·mL^−1^ vancomycin in a MACS-VA500 workstation (Don Whitley Scientific Ltd.) under microaerobic conditions (10% vol/vol O_2_, 5% vol/vol CO_2_, 85% N_2_, 42 °C). DNA was extracted using the QIAamp DNA Mini Kit (QIAGEN), according to manufacturer’s instructions and quantified on a Nanodrop spectrophotometer prior to normalization and sequencing. High-throughput sequencing was performed using an Illumina MiSeq benchtop sequencer (Illumina). Short read paired-end data were assembled using SPAdes (version 3.10.035) and evaluated using QUAST (76). All assembled genomes were uploaded to a local instance of the BIGSdb web-based database platform (77) which allowed for archiving and gene-by-gene sequence alignment. A total of 14 genomes with assembled length >1.9 Mbp, assembled in >500 contigs, and with an N_95_ < 800 bp were considered of poor quality and excluded from the analyses.

### Core and Accessory Genome Variation and Phylogenetic Reconstruction.

Sequence data were analyzed using a reference pan-genome approach (78) in which a list was compiled for all of the genes present in: 1) reference *C. jejuni* strains NCTC11168, 81116, 81–176, M1; 2) plasmids pTet and pVir; and 3) annotations from the 1,198 genomes in this study. Closely related homologous genes were identified using BLAST (>70% sequence identity) and filtered out to produce a single gene list containing all of the unique distinct genes for the dataset. Automatic annotations were obtained using RAST (79), and a total of 3,855 unique genes were described for the dataset from 1,967,096 open reading frames (ORFs). Gene orthologs were aligned in a gene-by-gene manner (78) using MAFFT (80) to produce a whole-genome multiple sequence alignment for all isolates of the dataset. The presence of individual gene sequences from the reference pan-genome list were detected in every genome of the dataset using BLAST with a match defined as >70% nucleotide identity over >50% of the gene length. This approach generated a gene presence/absence matrix summarizing the presence and allelic variation of every gene in every genome. The core genome was defined as genes shared by all isolates, while a “soft core” represented genes shared >95% by all isolates (16). The remaining genes constituted the accessory genome. Phylogenetic trees in analysis of gene-by-gene alignments of core genes (18), single-gene alignments, were reconstructed using the approximation of the ML algorithm implemented in RAxML v8.2.11 (81) with the GTRGAMMA model. PhyML v3.3.2 (82) and FastTree2 (83) with GTR model of nucleotide substitution were also used to reconstruct the ML phylogenetic trees that were used for recombination and time-scaled analyses, respectively. Tajima’s D was estimated over all sites which did not contain undetermined or missing bases using the PopGenome package (v2.6.1) in R (84, 85). Demographic reconstruction analysis was conducted using the nonparametric Bayesian Skyline model (86) in BEAST2 (21) and visualized with Tracer v1.7.1 (87). A GTR+G4 DNA substitution model was used in combination with a relaxed log-normal clock model and Coalescent Bayesian Skyline tree prior. A prior on the clock rate was set as a log-normal distribution with a mean value of 1 × 10^−6^ mutations per site per year (88).

### Detection of Recombination in *C. jejuni* ST-61 and ST-21 Complexes.

A subset of 99 isolates comprising 50 ST-61 complex isolates and 49 ST-21 complex isolates was used for further recombination analysis (Dataset S1). A reference pan-genome for this subset was created as described above. From a total of 174,730 ORFs, 2,570 genes remained after the removal of allelic variants. Of these, 1,498 genes were shared by 95% of all isolates, from which a gene-by-gene alignment was created using MAFFT (80). Homologous recombination events were inferred using ClonalFrameML (89) using a guiding phylogeny reconstructed with PhyML (82). The transition/transversion rate for ClonalFrameML was set to 8.093, a value calculated by PhyML. The resulting estimates were *R*/θ = 0.606459 (relative rate of recombination to mutation), *1*/δ = 0.00209515 (inverse mean DNA import length) and ν = 0.028986 (mean divergence of imported DNA). The prevalence of exactly similar recombination regions was individually assessed in all isolates using BLAST with a threshold of 100% sequence identity. We adopted the conservative approach of excluding recombination regions with a sequence length >1,000 bp, as these regions were likely to contain more than two genes and have reduced biological significance when using a concatenated gene-by-gene alignment as an input for this analysis. The relative number of substitutions introduced by recombination (*r*) and mutation (*m*) was calculated as the ratio *r*/*m* = (*R*/θ) × δ × ν, with parameters inferred directly from ClonalFrameML using the transition/transversion ratio values and a guiding phylogeny computed with PhyML.

### Detection of Homoplasy in Cattle-Adapted Lineages.

Homoplasy was defined as genetic similarity in divergent lineages that was absent in their common ancestor. Analysis was carried out to detect homoplasious accessory gene loss/gain and homologous recombination based on comparison of ST-61 complex isolates with ST-42, ST-21, and 11 other clonal complexes. Accessory gene presence was defined as a >70% BLAST match over 50% or more of any gene in the pan-genome list. For homologous recombination inferred in 49 ST-61 complex isolates using ClonalFrameML, recombinant sequence blocks (8 and 6,191 bp) were extracted from the alignment and sequence <1,000 bp (*n* = 140) was locally aligned using BLAST to 328 genomes. This reference genome set included isolates from cattle and chicken belonging to ST-21, ST-45, ST-42, ST-257, ST-353, ST-354, ST-443, ST-464, ST-573, ST-574, ST-607, and ST-661 complexes. An alignment match was considered homoplasious when there was an exact sequence match of 100% sequence identity and length found in two phylogenetically distinct lineages. Recombinant sequence >1,000 bp in length (*n* = 35) were analyzed using STRUCTURE software (9) to differentiate concatenates of different genes or gene fragments of genes in gene-by-gene alignments. Briefly, the No Admixture model with an independent allele frequency model was used to calculate the frequency of all alleles of 1,469 pan-genome loci that were present in the reference genome set. Analyses were performed with 100 burn-in cycles followed by 1,000 iterations. Homoplasies were identified for 77 isolates (Dataset S1) belonging to 13 clonal complexes using HomoplasyFinder (49). Briefly, gene orthologs were aligned in a gene-by-gene manner using MAFFT (80) to produce a whole-genome multiple sequence alignment. The core alignment (1,423 genes >90% present) was used to quantify homoplasies on branches leading to 13 ST complexes using HomoplasyFinder (49).

### Time-Scaled Phylogenetic Analyses.

The timescale of emergence and diversification of the cattle-associated ST-61 complex was estimated using 41 isolates with a known isolation date, with ST-5161 isolate as the outgroup. Core genes were aligned using MAFFT (80). Genes were then ordered using *C. jejuni* NCTC11168 and concatenated to generate an ordered core gene alignment. This core gene alignment was used to reconstruct a phylogeny using RAxML (81). Recombination regions, inferred by ClonalFrameML, were masked from the core genome sequence alignment (i.e., replaced with gaps) using a custom script (available on https://github.com/kwongj/cfml-maskrc), and the temporal signal was investigated with linear regression analysis of the root-to-tip distances against the sampling years using TempEst v1.5.1 (22). A time-scale phylogeny was constructed using BEAST2 v2.5.0 (21), based on 1,320 variable sites, using the GTR +G4 model of DNA substitution. We compared the marginal likelihood of nine different combinations of the strict clock, relaxed clock exponential, and relaxed clock log-normal models with the constant population, exponential population growth, and Bayesian skyline models using generalized stepping-stone sampling (90), run for 1 million iterations in 30 path steps. The relaxed log-normal clock with Bayesian skyline model showed the largest marginal likelihood. Input xml files were prepared using BEAUti2 v.2.5.0 (21). A prior on the clock rate was set as a log-normal distribution with a mean value of 1 × 10^−6^ mutations per site per year (88). Markov chains were run for 100 million generations, sampled every 10,000 generations with the first 10,000,000 generations (10%) discarded as burn-in. Three independent runs of 100 million generations were conducted and convergence was assessed by checking that the effective sample size of all parameters exceeded 400 using Tracer v1.7.1 (87). TreeAnnotator (implemented in BEAST2 package) was used to generate a maximum clade credibility tree after discarding 10% burn-in.

### Mutagenesis.

The gene block absent from cattle specialists (*cj1324-1332*) was inactivated in two randomly selected chicken specialist strains from distinct sequence clusters id69 (ST-661) and id424 (ST-257) (Dataset S1) by deletion of the majority of the ORFs and replacement with a kanamycin resistance cassette through allelic exchange mutagenesis. The mutation vector was created using the Gibson isothermal assembly method as described previously (91) using primers geneblockF1/R1 and F2/R2 to amplify upstream and downstream regions flanking the deletion and KanF and KanR primers to amplify the resistance cassette (*SI Appendix*, Table S2). The fragments were assembled into HincII digested pGEM3Zf to form pBLOCK. Competent cells of *C. jejuni* strains id69 and id424 were prepared by washing in cold 9% (wt/vol) sucrose, 15% (vol/vol) glycerol solution, and were transformed with pBLOCK by electroporation. Mutant clones were selected by kanamycin resistance and correct insertion of the kanamycin cassette into the genome confirmed by PCR screening with primers geneblockF1 and geneblockR2 (*SI Appendix*, Table S2). The ΔGB (gene block) strains were screened in phenotype assays along with 36 *C. jejuni* isolates from cattle specialist ST-61 (*n* = 6) and ST-42 (*n* = 6) complexes, chicken specialist ST-257 (*n* = 5), ST-353 (*n* = 1), ST-573 (*n* = 2), ST-574 (*n* = 2), ST-607 (*n* = 1), and ST-661 (*n* = 1) complexes, as well as the generalist ST-21 (*n* = 12) complex isolates of cattle, chicken, and clinical origin (Dataset S1).

### Cell Surface Hydrophobicity Assay.

Overnight growth was harvested from agar plates and resuspended in 2 mM Na phosphate buffer (pH 7.4) to an optical density at 600 nm of ∼1.0. Twenty microliters of cell suspension was aliquoted per well in U-bottomed 96-well plates (Greiner BioOne 650161) and 180 µL of a two-fold serial dilution of ammonium sulfate (2 M to 0.98 mM) added. Plates were incubated statically at room temperature for 3 d. The minimum concentration of ammonium sulfate permitting aggregation of cells defines the point of hydrophobicity (51).

### Auto-Agglutination Assay.

Overnight growth was harvested from agar plates and resuspended in PBS to an optical density at 600 nm of ∼1.0. An amount of 1 mL of the suspension was sampled, and an accurate starting optical density was measured. Cell suspensions were then incubated under standard microaerobic conditions statically in plastic tubes for 1.5 h. The top 1 mL of the suspension was then carefully removed and the optical density measured. The percentage (%) of autoagglutination was determined by subtracting the OD_600_ measured after 1.5 h from the starting OD_600_, dividing by the starting OD_600_ and multiplying by 100 [(Starting OD_600_ − Final OD_600_)/Starting OD_600_) × 100] (51).

### Biofilm Assay.

Overnight growth was harvested from agar plates and resuspended in Muller Hinton broth to an optical density at 600 nm of ∼0.2. Two hundred microliters of the cell suspension was aliquoted into 12 replicate wells of a tissue culture treated 96-well plate (CellStar 655180). Plates were incubated under standard microaerobic conditions statically for 3 d. The culture was removed and wells stained with 1% (wt/vol) crystal violet in 90% (vol/vol) ethanol for 5 min. The stain was removed and wells washed three times with dH_2_O. Biofilms were apparent as clearly stained rings corresponding to the liquid-air interface. Plates were then allowed to air dry before destaining with 300 µL of 80% vol/vol ethanol, 20% vol/vol acetone for 10 min. One hundred microliters was transferred per well to an optically clear 96-well plate and the optical density measured at 570 nm in a SpectraMax plate reader (Molecular Devices). Muller Hinton broth controls were performed to normalize plate-to-plate variation.

### Data Availability.

Short-read data for genomes sequenced as part of this study are archived on the National Center for Biotechnology Information Short Read Archive associated with BioProject accession no. PRJNA575343. Contiguous assemblies of all genome sequences compared are available at the public data repository Figshare (DOI: 10.6084/m9.figshare.9929054). Individual accession numbers can be found in Dataset S1.

## Supplementary Material

Supplementary File

Supplementary File

Supplementary File

Supplementary File
