# Supplementary material for: Agricultural intensification and the evolution of host specialism in the enteric pathogen Campylobacter jejuni
Source: Proc Natl Acad Sci U S A. 2020 May 4;117(20):11018–28. doi: 10.1073/pnas.1917168117 (PMC7245135; doi:10.1073/pnas.1917168117)
Supplement: Supplementary File [file pnas.1917168117.sapp.pdf]

Supplementary Information for

Agricultural intensification and the evolution of host specialism in the enteric pathogen *Campylobacter jejuni*.

Evangelos Mourkas, Aidan J. Taylor, Guillaume Méric, Sion C. Bayliss, Ben Pascoe, Leonardos Mageiros, Jessica K. Calland, Matthew D. Hitchings, Anne Ridley, Ana Vidal, Ken J. Forbes, Norval J. C. Strachan, Craig T. Parker, Julian Parkhill, Keith A. Jolley, Alison J. Cody, Martin C. J. Maiden, David J. Kelly*, Samuel K. Sheppard*.

*Corresponding authors: Samuel K. Sheppard; and David J. Kelly;
Email: s.k.sheppard@bath.ac.uk, d.kelly@sheffield.ac.uk

This PDF file includes:

Supporting Figure, Table and Dataset legends
Figures S1 to S9
Tables S1 and S2
SI References

www.pnas.org/cgi/doi/10.1073/pnas.1917168117

Figure S1. Effective population size of cattle specialists ST-61 and ST-42 phylogenies over time. The plot displays the effective population size and the generation length in years (Ne) of the ST-61 (A) and ST-42 (B) lineages in relation to their phylogenies. The solid blue line shows the median effective population size over time, while the shaded blue colored area indicates the 95% Highest Posterior Density. The effective population size (Ne) is shown on the *y* axis and the timeframe in years between the most recent sampling date and the emergence of each lineage on the *x* axis. The gray line indicates the line to the most recent common ancestor shared with the outgroup (MRCA).

Figure S2. Distribution of genes that harbour ST-21 (white) or ST-61 (black) specific alleles. The number of genes is illustrated in the *y* axis while the ST-21 and ST-61 complexes as well as the intermediates ST38 and ST5161 in the *x* axis.

Figure S3. Phylogenetic tree structure of all 1,198 *C. jejuni* isolates and gene presence /absence matrix per isolate.

Figure S4. Number of genes of all ST-61 isolates. The total number of genes for every isolate is illustrated in the *y* axis, with isolation date on the *x* axis.

Figure S5. Prevalence of ST-61 complex alleles within the core genome of multiple lineages. The average number of ST-61 alleles per isolate (*y* axis) is calculated for all clonal complexes with isolates sampled from both cattle (blue) and chicken (yellow).

Figure S6. All recombination events as inferred by ClonalFrameML. The number of recombination events is plotted against the length (bp) of the recombinant sequence.

Figure S7. Homoplasies identified in 13 *C. jejuni* clonal complexes. Number of homoplasies identified using HomoplasyFinder is shown for 13 clonal complexes.

Figure S8. Phylogenetic structure of 381 *C. jejuni* isolates of cattle, sheep and chicken isolates and the distribution of the candidate cattle-associated genes and their alleles. Left panel: All genome sequences were from ST-61 (blue), ST-21 (yellow), ST-45 (black), ST-42 (light blue), ST-257 (black), ST-353 (black), ST-354 (black), ST-443 (black), ST-464, ST-573 (black), ST-574, ST-607 (black) and ST-661 (black) complexes. Right panel: Each bar indicates the presence of a gene for each isolate while the difference in color corresponds to different allele of the same gene. The red box highlights a block of genes absent in ST-61 and ST-42 complexes. The phylogenetic trees were reconstructed using an approximation of the maximum-likelihood algorithm in RAxML.

Figure S9. Prevalence of ST-61-42 complex homoplasy in 13 *C. jejuni* clonal complexes. (A) Prevalence of homoplasious genes gained (upper plot) and that are lost (bottom plot) in ST-61 and in 12 other *C. jejuni* ST-complexes with isolates sampled from both cattle (blue) and chicken (yellow). (B) Prevalence of homoplasious recombination in ST-61 and in 12 other *C. jejuni* ST-complexes with isolates sampled from both cattle (blue) and chicken (yellow). Most homoplasies were largely restricted to the cattle specialist ST-61 and ST-42 complexes.

Table S1. Recombination and transition/transversion parameters as calculated by PhyML and CFML.

Table S2. Primers and their corresponding sequences used for cloning and mutagenesis confirmation. The uppercase sequences of the geneblock primers are the adaptor regions used in the Gibson assembly cloning, while the lowercase sequences are the regions annealing to a region upstream of *cj1324* (F1) and just inside the *cj1324* coding region (R1) or towards the end of the *cj1332* coding region (F2) and downstream of *cj1332* (R2). The KanF and KanR primers are adaptors that also amplify the kan gene from pJMK30 (120).

Dataset S1. Isolate information about the *C. jejuni* strains used in this study.

Dataset S2. Prevalence in 13 *C. jejuni* clonal complexes of 301 genes identified as being distinctly associated with the emergence of ST-61 complex isolates from ST-21 clonal complex.

Dataset S3. Genetic elements associated with ST-42 complex cattle specialization.

Fig. S1

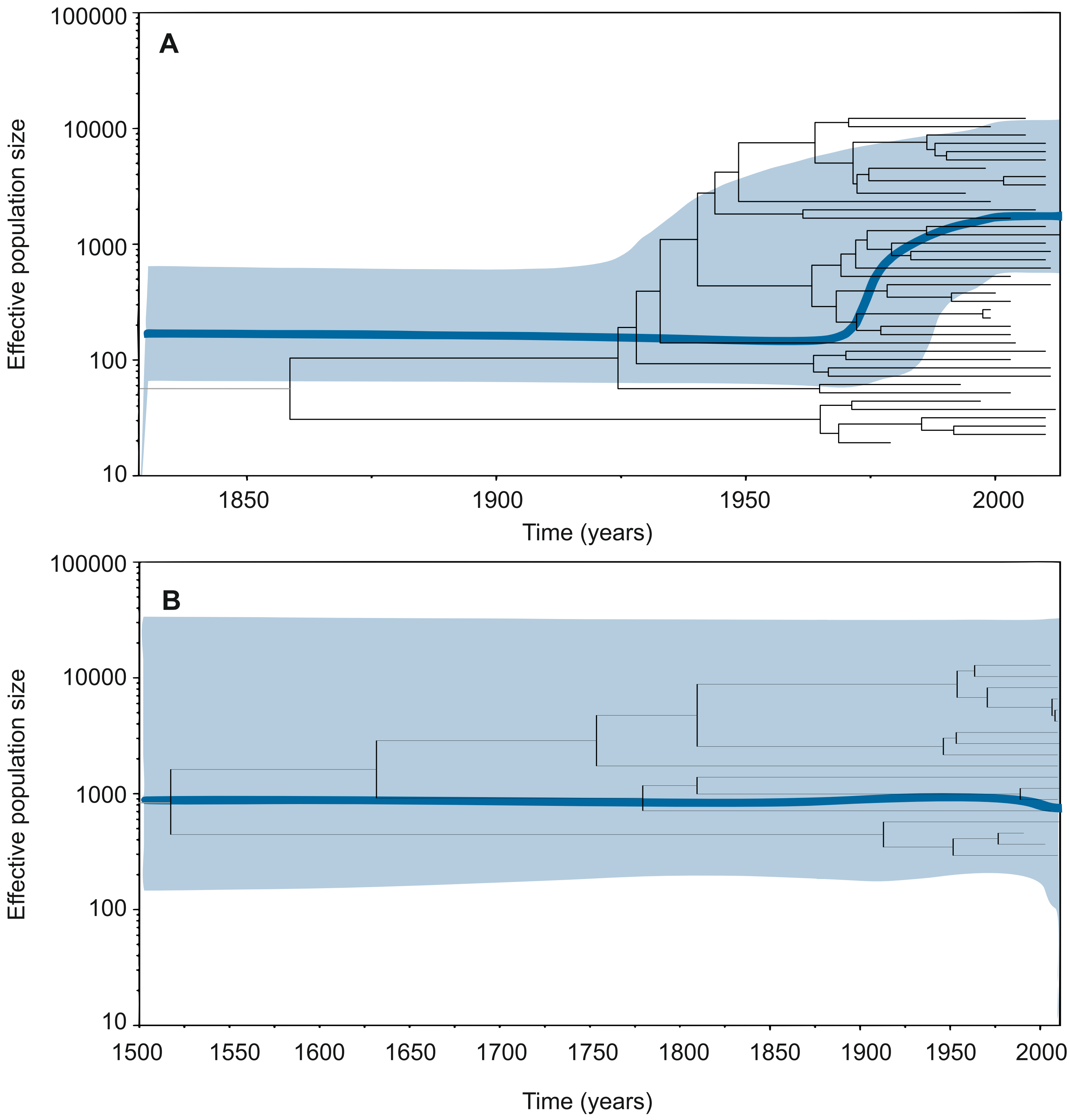
A
B
100000
10000
1000
100
10
Effective population size
1850
1900
1950
2000
1500
1550
1600
1650
1700
1750
1800
1850
1900
1950
2000
Time (years)

Fig. S2

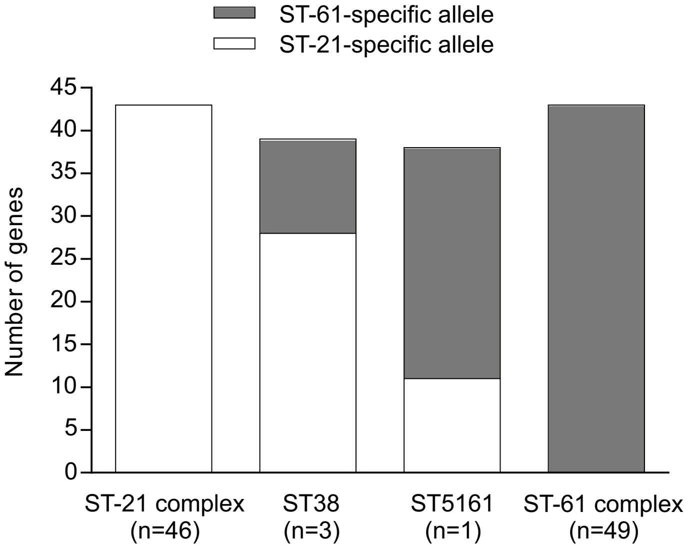
ST-61-specific allele
ST-21-specific allele
Number of genes
45
40
35
30
25
20
15
10
5
0
ST-21 complex (n=46)
ST38 (n=3)
ST5161 (n=1)
ST-61 complex (n=49)

Fig. S3

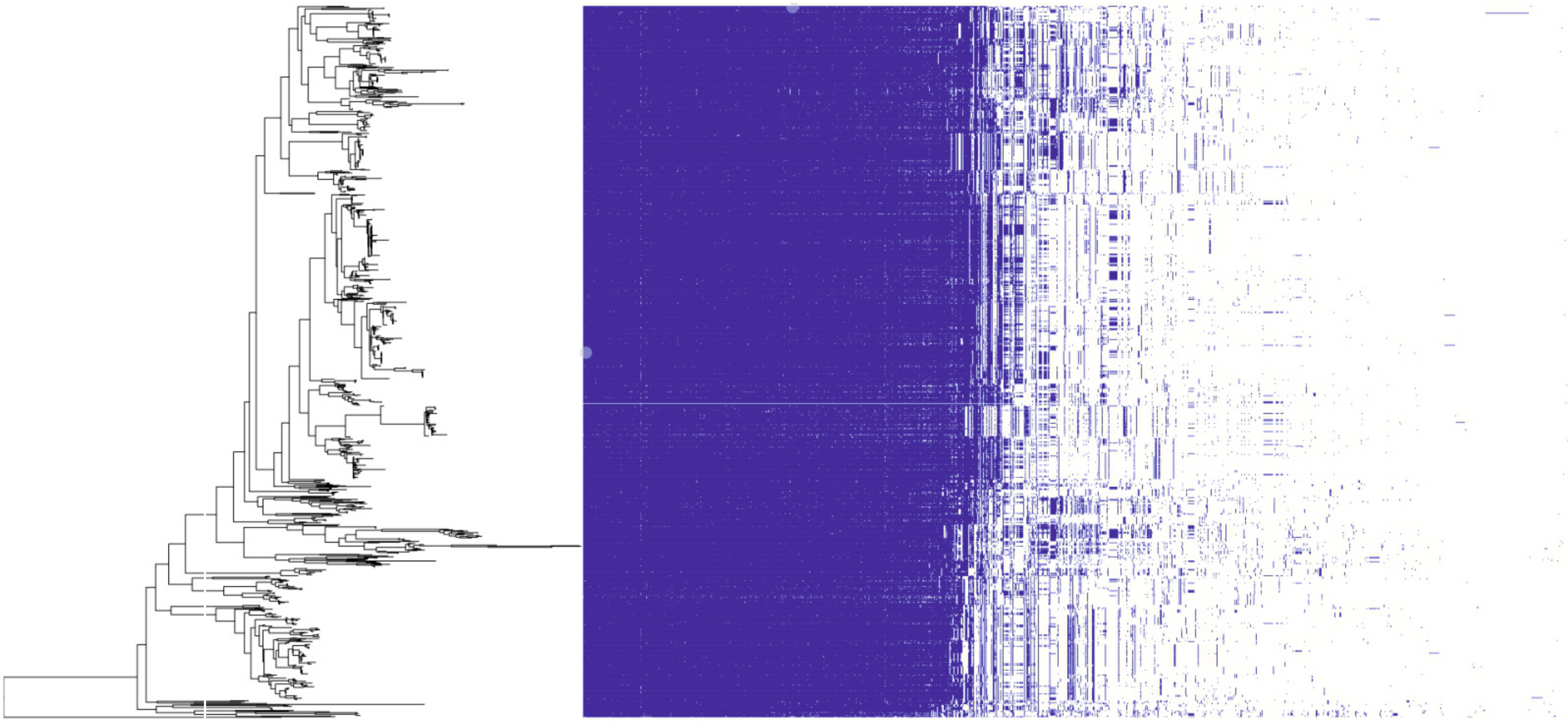

Fig. S4

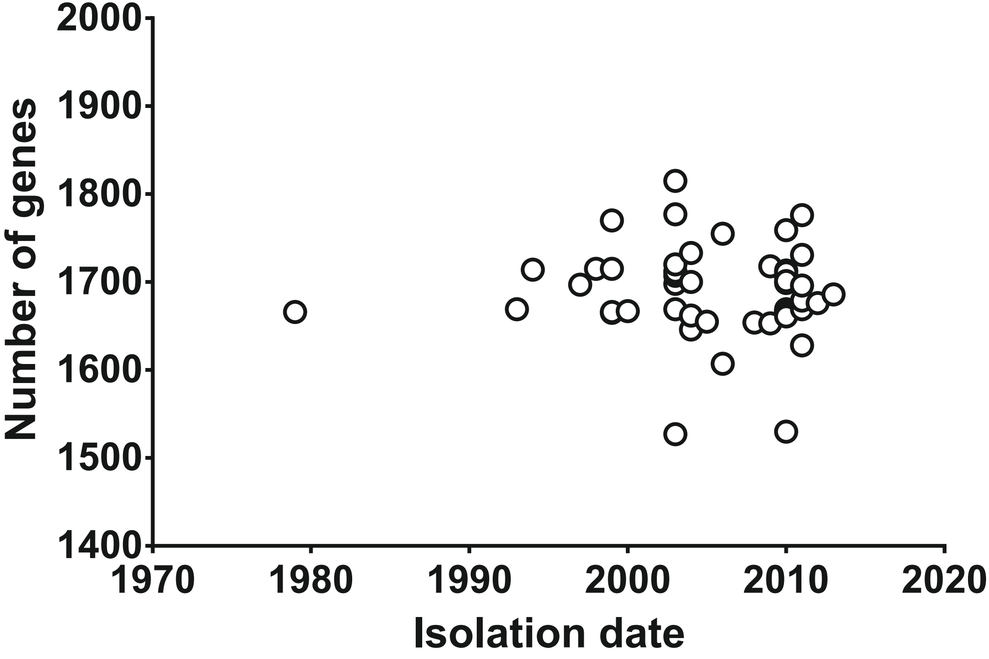

Number of genes
2000
1900
1800
1700
1600
1500
1400
1970
1980
1990
2000
2010
2020
Isolation date

Fig. S5

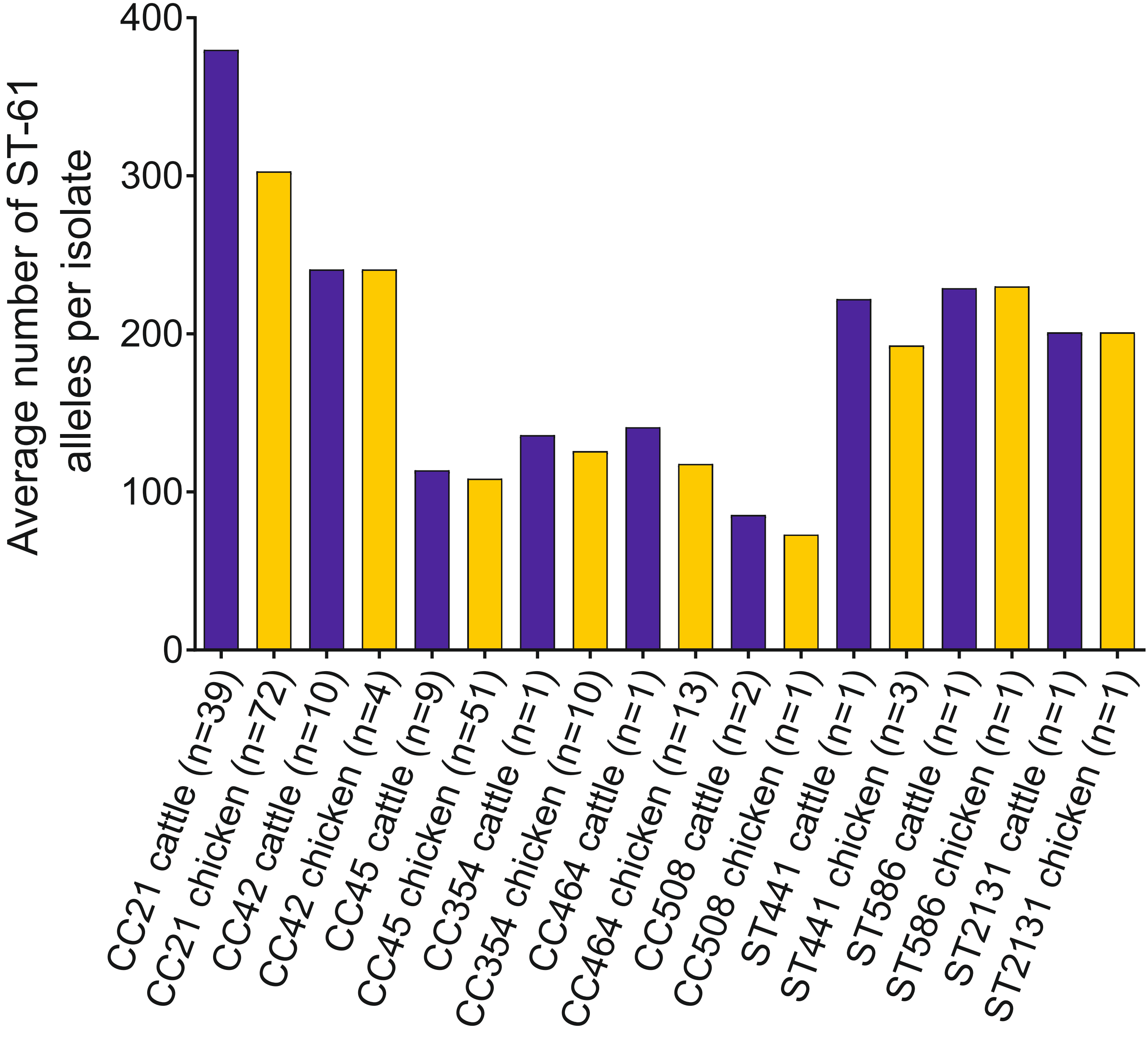

Average number of ST-61 alleles per isolate
0
100
200
300
400
CC21 cattle (n=39)
CC21 chicken (n=72)
CC42 cattle (n=10)
CC42 chicken (n=4)
CC45 cattle (n=9)
CC45 chicken (n=51)
CC354 cattle (n=1)
CC354 chicken (n=10)
CC464 cattle (n=1)
CC464 chicken (n=13)
CC508 cattle (n=2)
CC508 chicken (n=1)
ST441 cattle (n=1)
ST441 chicken (n=3)
ST586 cattle (n=1)
ST586 chicken (n=1)
ST2131 cattle (n=1)
ST2131 chicken (n=1)

Fig. S6

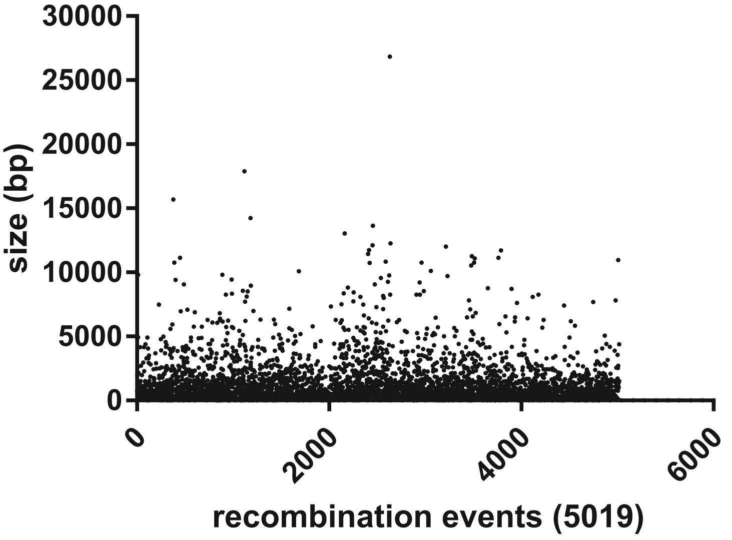
30000
25000
20000
15000
10000
5000
0
size (bp)
0
2000
4000
6000
recombination events (5019)

Fig. S7

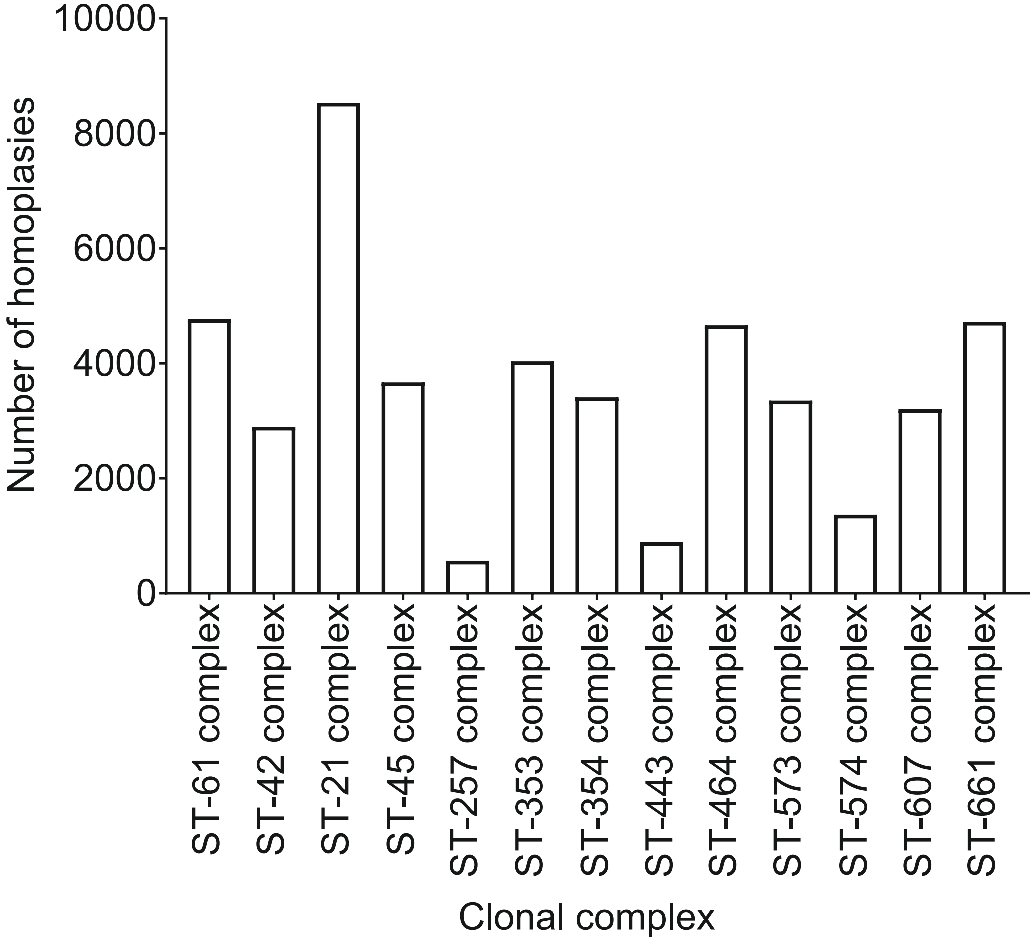

10000
8000
6000
4000
2000
0
Number of homoplasies
ST-61 complex
ST-42 complex
ST-21 complex
ST-45 complex
ST-257 complex
ST-353 complex
ST-354 complex
ST-443 complex
ST-464 complex
ST-573 complex
ST-574 complex
ST-607 complex
ST-661 complex
Clonal complex

Fig. S8

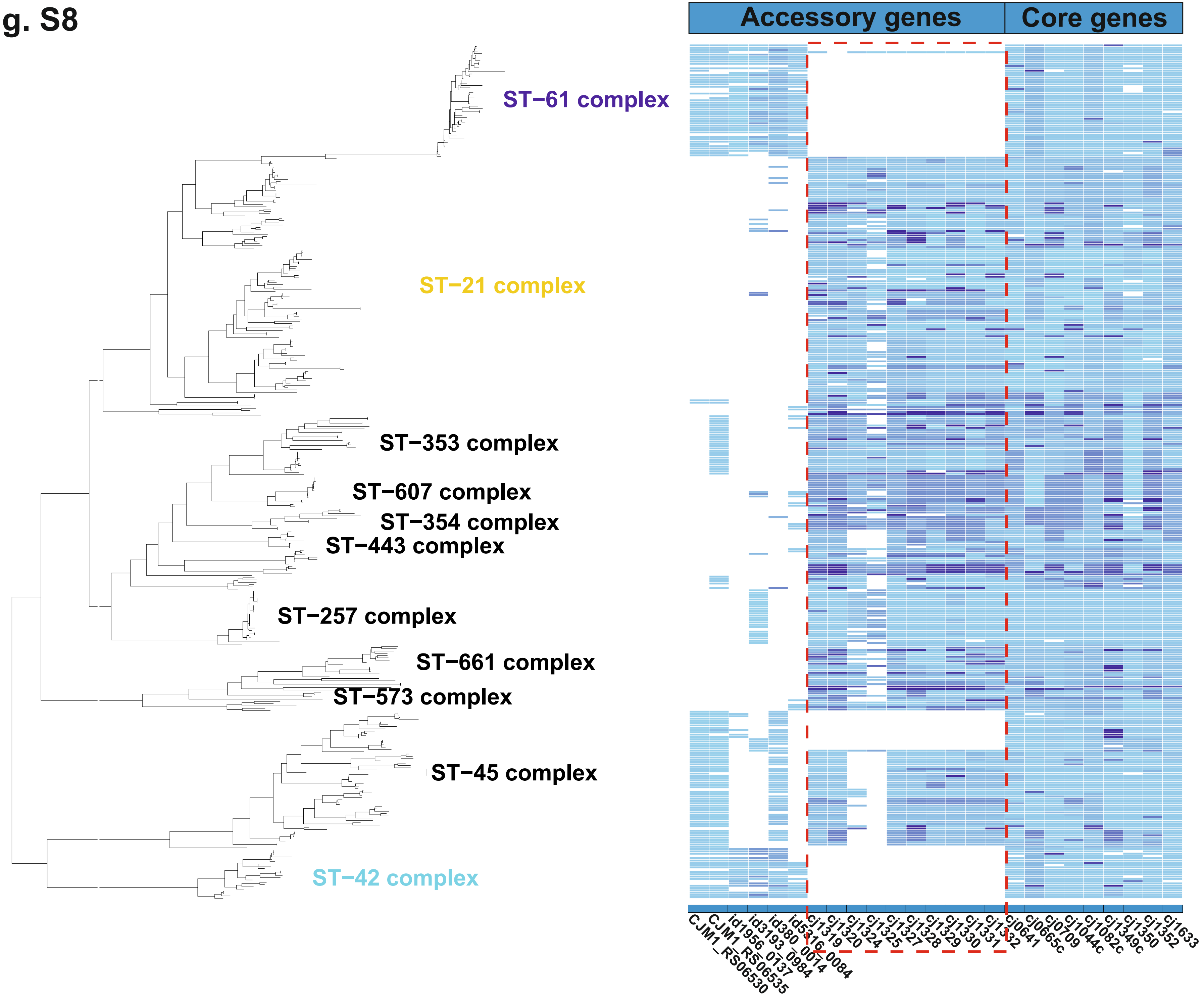
Accessory genes
Core genes
ST−61 complex
ST−21 complex
ST−353 complex
ST−607 complex
ST−354 complex
ST−443 complex
ST−257 complex
ST−661 complex
ST−573 complex
ST−45 complex
ST−42 complex
CJM1_RS06530
CJM1_RS06535
id1956_0137
id3193_0984
id380_0014
id5316_0084
cj1319
cj1320
cj1324
cj1325
cj1327
cj1328
cj1329
cj1330
cj1331
cj1332
cj0641
cj0665c
cj0709
cj1044c
cj1082c
cj1349c
cj1350
cj1352
cj1633

Fig. S9

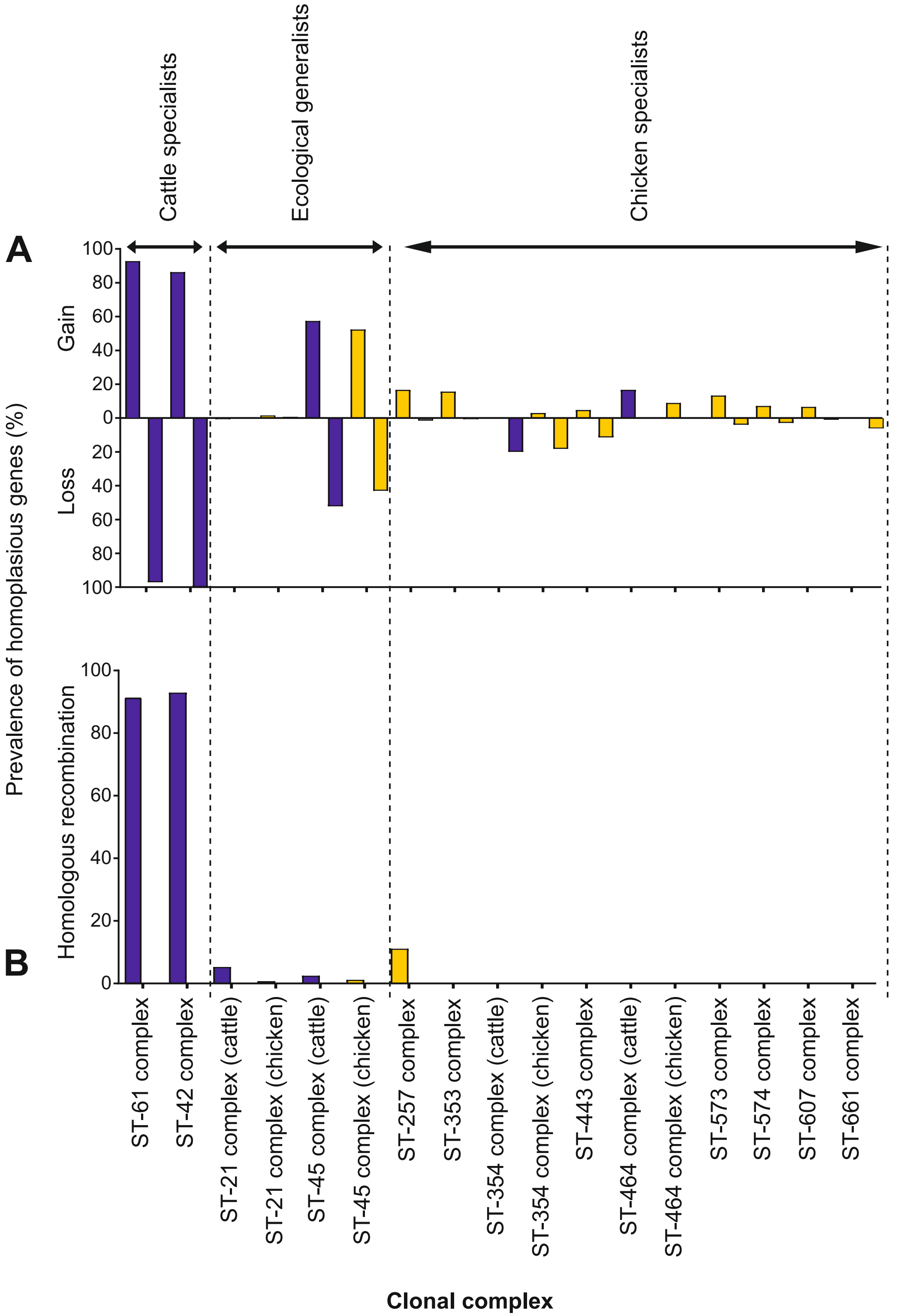

Cattle specialists
Ecological generalists
Chicken specialists
A
B
Prevalence of homoplasious genes (%)
Gain
Loss
Homologous recombination
100
80
60
40
20
0
ST-61 complex
ST-42 complex
ST-21 complex (cattle)
ST-21 complex (chicken)
ST-45 complex (cattle)
ST-45 complex (chicken)
ST-257 complex
ST-353 complex
ST-354 complex (cattle)
ST-354 complex (chicken)
ST-443 complex
ST-464 complex (cattle)
ST-464 complex (chicken)
ST-573 complex
ST-574 complex
ST-607 complex
ST-661 complex
Clonal complex

Table S1. Recombination and transition/transversion parameters. Values are calculated by PhyML and ClonalFrameML

Clonal complex	n	transition/transversion	R/θ	1/δ	v	r/m = R/θ*δ*v
ST-21 complex	49	8.074	0.620008	0.00246347	0.0297719	7.493014396
ST-61 complex	50	4.295	0.590489	0.00216759	0.0462289	12.59355179
ST-21 / ST-61 subset used in ClonalFrame ML	99	8.093	0.606459	0.00209515	0.028986	8.390244409
Whole dataset	1198	-	0.644303	0.00127492	0.0189957	9.599807437

Table S2. Primers and their corresponding sequences used for cloning and mutagenesis confirmation. The uppercase sequences of the geneblock primers are the adaptor regions used in the Gibson assembly cloning, while the lowercase sequences are the regions annealing to a region upstream of *cj1324* (F1) and just inside the *cj1324* coding region (R1) or towards the end of the *cj1332* coding region (F2) and downstream of *cj1332* (R2). The KanF and KanR primers are adaptors that also amplify the *kan* gene from pJMK30.

Primer name	Sequence 5' – 3'
geneblockF1	GAGCTCGGTACCCGGGGATCCTCTAGAGTCgataaataccgcagaatgaat
geneblockR1	AAGCTGTCAAACATGAGAACCAAGGAGAATcacgacttttagcaaaaatataa
geneblockF2	GAATTGTTTTAGTACCTAGCCAAGGTGTGCtttgtagcaggagcttgtg
geneblockR2	AGAATACTCAAGCTTGCATGCCTGCAGGTCatgttttgattaaaaagctcttt
KanF	ATTCTCCTTGGTTCTCATGTTTGACAGCTTAT
KanR	GCACACCTTGGCTAGGTACTAAAACAATTCAT

References

1. Atterby C, et al. (2018) The Potential of Isolation Source to Predict Colonization in Avian Hosts: A Case Study in *Campylobacter jejuni* Strains From Three Bird Species. *Front Microbiol* 9(March):1–12.
2. Baily JL, et al. (2015) Evidence of land-sea transfer of the zoonotic pathogen *Campylobacter* to a wildlife marine sentinel species. *Mol Ecol* 24(1):208–221.
3. Biggs PJ, et al. (2011) Whole-Genome Comparison of Two *Campylobacter jejuni* Isolates of the Same Sequence Type Reveals Multiple Loci of Different Ancestral Lineage. *PLoS One* 6(11):e27121.
4. Loc Carrillo C, et al. (2005) Bacteriophage Therapy To Reduce *Campylobacter jejuni* Colonization of Broiler Chickens. *Appl Environ Microbiol* 71(11):6554–6563.
5. Cody AJ, et al. (2013) Real-Time Genomic Epidemiological Evaluation of Human *Campylobacter* Isolates by Use of Whole-Genome Multilocus Sequence Typing. *J Clin Microbiol* 51(8):2526–2534.
6. Cooper KK, Cooper MA, Zuccolo A, Law B, Joens LA (2011) Complete Genome Sequence of *Campylobacter jejuni* Strain S3. *J Bacteriol* 193(6):1491–1492.
7. Gripp E, et al. (2011) Closely related *Campylobacter jejuni* strains from different sources reveal a generalist rather than a specialist lifestyle. *BMC Genomics* 12(1):584.
8. Gunther NW, Bono JL, Needleman DS (2015) Complete Genome Sequence of *Campylobacter jejuni* RM1285, a Rod-Shaped Morphological Variant. *Genome Announc* 3(6):e01361-15.
9. He Y, et al. (2015) Complete Genome Sequence of *Campylobacter jejuni* YH001 from Beef Liver, Which Contains a Novel Plasmid. *Genome Announc* 3(1):7–8.
10. Morley L, et al. (2015) Gene Loss and Lineage-Specific Restriction-Modification Systems Associated with Niche Differentiation in the *Campylobacter jejuni* Sequence Type 403 Clonal Complex. *Appl Environ Microbiol* 81(11):3641–3647.
11. Mourkas E, et al. (2019) Gene pool transmission of multidrug resistance among *Campylobacter* from livestock, sewage and human disease. *Environ Microbiol* 00:1462-2920.14760.
12. Pascoe B, et al. (2017) Local genes for local bacteria: Evidence of allopatry in the genomes of transatlantic *Campylobacter* populations. *Mol Ecol* 26(17):4497–4508.
13. Richards VP, Lefébure T, Pavinski Bitar PD, Stanhope MJ (2013) Comparative characterization of the virulence gene clusters (lipooligosaccharide [LOS] and capsular polysaccharide [CPS]) for *Campylobacter coli*, *Campylobacter jejuni* subsp. *jejuni* and related *Campylobacter* species. *Infect Genet Evol* 14(1):200–213.
14. Sheppard SK, et al. (2014) Cryptic ecology among host generalist *Campylobacter jejuni* in domestic animals. *Mol Ecol* 23(10):2442–2451.
15. Sheppard SK, et al. (2013) Progressive genome-wide introgression in agricultural *Campylobacter coli*. *Mol Ecol* 22(4):1051–1064.
16. Sheppard SK, et al. (2013) Genome-wide association study identifies vitamin B5 biosynthesis as a host specificity factor in *Campylobacter*. *Proc Natl Acad Sci* 110(29):11923–11927.
17. Takamiya M, et al. (2011) Genome Sequences of Two Stress-Tolerant *Campylobacter jejuni* Poultry Strains, 305 and DFVF1099. *J Bacteriol* 193(19):5546–5547.
18. Takamiya M, et al. (2011) Genome Sequence of *Campylobacter jejuni* strain 327, a strain isolated from a turkey slaughterhouse. *Stand Genomic Sci* 4(2):113–122.
19. Teh AHT, Lee SM, Dykes GA (2016) Draft Genome Sequences of Three Multiantibiotic-Resistant *Campylobacter jejuni* Strains (2865, 2868, and 2871) Isolated from Poultry at Retail Outlets in Malaysia. *Genome Announc* 4(3):e00331-16.
20. Thépault A, et al. (2017) Genome-Wide Identification of Host-Segregating Epidemiological Markers for Source Attribution in *Campylobacter jejuni*. *Appl Environ Microbiol* 83(7):e03085-16.
21. Yahara K, et al. (2016) Genome-wide association of functional traits linked with *Campylobacter jejuni* survival from farm to fork. *Environ Microbiol*:2–41.
22. Zeng X, Mo Y, Xu F, Lin J (2013) Identification and characterization of a periplasmic

trilactone esterase, Cee, revealed unique features of ferric enterobactin acquisition in *Campylobacter*. *Mol Microbiol* 87(3):594–608.

23. Jolley KA, Bray JE, Maiden MCJ (2018) Open-access bacterial population genomics: BIGSdb software, the PubMLST.org website and their applications. *Wellcome Open Res* 3(0):124.
24. Taylor AJ, Zakai SAI, Kelly DJ (2017) The Periplasmic Chaperone Network of *Campylobacter jejuni*: Evidence that SalC (Cj1289) and PpiD (Cj0694) Are Involved in Maintaining Outer Membrane Integrity. *Front Microbiol* 8(MAR):1–14.
